# The relationship between interdisciplinary learning motivation and learning burnout among university music majors in the context of AI-assisted music education: the mediating role of perceived learning load

**DOI:** 10.3389/fpsyg.2026.1922731

**Published:** 2026-08-07

**Authors:** Ya Zhang

**Affiliations:** Editorial Department of the Journal, Nanyang Institute of Technology, Nanyang, China

**Keywords:** AI-assisted music education, interdisciplinary learning motivation, learning burnout, perceived learning load, university music majors

## Abstract

**Introduction:**

University music majors increasingly encounter AI-related learning tasks while managing instrumental or vocal practice, ensemble rehearsals, theory coursework, performance assessment, and teaching preparation. This study examined the relationships among autonomous interdisciplinary motivation, pressure-driven interdisciplinary motivation, perceived learning load, and learning burnout in this combined learning context.

**Methods:**

Anonymous cross-sectional questionnaire data were collected from a convenience sample of 237 first- to third-year music majors at one university. The data were analyzed using reliability estimation, confirmatory factor analysis, Pearson correlation analysis, hierarchical regression, and Bootstrap testing with 5,000 resamples.

**Results:**

Autonomous interdisciplinary motivation was associated with lower learning burnout, whereas pressure-driven interdisciplinary motivation was associated with higher learning burnout. Perceived learning load accounted for part of the association between pressure-driven interdisciplinary motivation and learning burnout, indicating a significant cross-sectional indirect effect through students' appraisal of time, energy, task density, and competence demands. Autonomous and pressure-driven interdisciplinary motivation was nearly independent in the sample. The four-factor measurement model also provided preliminary support for treating autonomous motivation, pressure-driven motivation, perceived learning load, and learning burnout as distinct constructs.

**Discussion:**

The findings suggest that the psychological consequences of AI-related learning among university music majors depend not only on exposure to AI tools but also on students' reasons for participation and their appraisal of the resulting learning demands. Supporting autonomous engagement and managing additional learning load may help reduce burnout in AI-assisted music education.

## 1 Introduction

University music majors work under learning demands that accumulate across daily vocal or instrumental practice, studio lessons, ensemble rehearsals, stage performances, theory coursework, ear training, composition assignments, pedagogy courses, teaching preparation, and repeated course assessment. These activities require long hours of technical correction, public evaluation, and gradual professional identity formation. Students learn to judge sound, technique, expression, and audience response while also preparing for competitions, internships, postgraduate study, and employment. Research on music learning and digital music education has shown that such training is shaped by creative practice, participation in musical communities, and evolving standards of musicianship (Webster, 2002; Bauer, 2014; Ruthmann and Mantie, 2017; Cain, 2004; Folkestad, 2006; Tobias, 2013). AI-related tasks add digital arrangement, automated feedback interpretation, audio editing, and technology-supported teaching preparation to these ordinary demands.

AI-assisted music education changes the work students do with musical material. AI-assisted composition, intelligent accompaniment, audio processing, digital arrangement, automated feedback, music analysis, and technology-supported instructional design ask students to generate alternatives, compare outputs, edit sound, interpret algorithmic suggestions, and decide whether a technologically produced result is musically convincing. Prior work has shown that AI and digital technologies can expand musical material generation, feedback channels, and creative toolchains (Zhang et al., 2024; Sanchez-Jara et al., 2024; Peng and Ratnavelu, 2024; Ma and Wang, 2025). In university programs, students increasingly evaluate competence through the combined handling of musical judgment, DAW operation, MIDI editing, prompt design, feedback interpretation, and classroom material preparation. Teacher guidance, institutional resources, previous technology experience, and broader higher-education policies further shape how students encounter these tasks (Zawacki-Richter et al., 2019; Holmes et al., 2019; Luckin et al., 2016; UNESCO, 2023).

Students may participate in AI-related learning for different reasons even when their observable activity looks similar. Autonomous interdisciplinary motivation (AM) refers to engagement with AI, digital music, music technology, or educational technology because students connect these areas with musical expression, creative exploration, professional expansion, and self-development. Pressure-driven interdisciplinary motivation (PM) refers to engagement shaped by course requirements, peer comparison, employment pressure, concerns about technological replacement, or changing professional standards. Self-determination theory links motivational quality to perceived control, persistence, and learning experience (Deci and Ryan, 1985; Ryan and Deci, 2000, 2017). A music major may enjoy using AI to test a harmonic idea while also worrying that weak digital skills will reduce future competitiveness, so AM and PM need to be measured as distinct sources of participation.

Perceived learning load (LL) captures students' appraisal of total learning capacity when traditional music study and AI- or digital music-related tasks occur together. The construct refers to perceived shortages of time and energy, the density of assignments, and the sense that new competence requirements are being added to practice, rehearsal, theory coursework, collaborative projects, digital audio editing, competition preparation, and career planning. Learning burnout (BO) refers to exhaustion, detachment from learning, and a reduced sense of accomplishment during music-related and interdisciplinary study. When AI learning is pursued mainly to avoid falling behind, students may experience software learning, audio editing, and AI-supported assignments as additional demands competing with practice and coursework.

Research on AI-assisted music education has described tools, learning resources, feedback functions, and creative possibilities, yet fewer studies have examined why music majors engage in AI-related learning and how that engagement is associated with load and burnout under high-intensity professional training. Recent higher-education studies likewise report AI anxiety, ethical anxiety, technostress, and academic anxiety among student users of generative AI (Zhan et al., 2023; Zhu et al., 2025; Cengiz and Peker, 2025; Dizon et al., 2025; Gámez-Guadix and Mateos-Pérez, 2026). Using anonymous questionnaire data from 237 1st- to 3rd-year university music majors, the present analysis examines whether AM and PM are related to BO in different directions and whether LL carries an indirect association between PM and BO. The analysis tests whether the two motivational sources show opposite associations with burnout and whether perceived learning load explains part of the pressure-driven path.

## 2 Related work and hypotheses

### 2.1 AI-assisted music education and changes in learning tasks

AI assistance changes music learning first at the level of musical material. Boden (1998) conceptualized computational creativity as the production and evaluation of novel combinations, a distinction that fits AI-assisted composition when students compare generated alternatives with their own musical intentions. Generative music systems widen the range of melodic, harmonic, timbral, and arrangement materials available for student selection and revision (Briot et al., 2020). In a composition or arrangement assignment, students therefore work with both the musical idea and the digital procedure that produced it. AI-assisted composition and music-learning platforms also expand the sources from which students receive examples, accompaniment drafts, and analytic prompts (Zhang et al., 2024; Sanchez-Jara et al., 2024; Peng and Ratnavelu, 2024).

Bauer (2014) framed digital music learning through creating, performing, responding, and connecting, which helps locate AI tools inside familiar music-education activities. Automated feedback and digital audio environments redirect students' attention toward editing, mixing, sequencing, sound comparison, and revision decisions (Webster, 2002; Ruthmann and Mantie, 2017). Intelligent accompaniment and audio-processing tools can support practice and production, yet the student still has to judge phrasing, balance, style, and expressive fit. For music education majors, AI-supported teaching tools also affect how listening materials, accompaniment tracks, classroom activities, and teaching demonstrations are prepared (Holmes et al., 2019; UNESCO, 2023).

Course assessment increasingly asks students to explain why an AI-assisted output serves a musical purpose and how a digital procedure was selected or revised. AI-related tasks in higher education depend on platform access, teacher support, institutional policy, and students' prior experience with educational technology (Zawacki-Richter et al., 2019; Williamson, 2017). Music curricula still form professional identity through studio practice, project work, performance, and participation in musical communities. Once AI-assisted tasks enter coursework, musical judgment, technical operation, and course evaluation become mutually dependent parts of the same learning activity.

### 2.2 Dual sources of interdisciplinary learning motivation

The same AI-related learning behavior can arise from different reasons for participation. Ryan and Deci (2000, 2017) distinguished autonomous regulation from controlled regulation by focusing on whether action is experienced as personally endorsed or pressured by external demands. Behavioral effort alone cannot show whether a music major is using AI-assisted composition to pursue a musical idea, satisfy a course requirement, keep up with peers, or prepare for future employment (Deci and Ryan, 1985; Vallerand et al., 1992). AM and PM are therefore treated as distinct motivational sources because a student can experience ownership and pressure during the same AI-related task.

Autonomous interdisciplinary motivation (AM) describes engagement with AI, digital music, music technology, or educational technology when students connect the activity with music-making and professional growth. Task value, interest development, and self-regulated learning help explain why students continue with activities they regard as useful, expressive, and personally meaningful (Eccles and Wigfield, 2002; Hidi and Renninger, 2006; Pintrich, 2003). AI-assisted composition, digital arrangement, audio editing, intelligent accompaniment, and teaching-material preparation can support AM when students use them to refine a sound, test an arrangement, prepare a lesson, or extend a creative project.

Venkatesh et al. (2003) positioned performance expectancy and social influence as central conditions for technology adoption, which is relevant when AI competence becomes visible in courses, competitions, internships, and employment preparation. Perceived usefulness can encourage music students to learn AI tools, while course evaluation and peer comparison can turn the same activity into a pressure-oriented obligation (Davis, 1989; Venkatesh et al., 2003). Fear of AI, ethical concern, and generative-AI anxiety add another layer of pressure when students worry about competence, risk, or future standing (Zhan et al., 2023; Zhu et al., 2025; Cengiz and Peker, 2025). Academic confidence and anxiety in generative-AI use further show that student engagement with AI can combine practical interest with unease about performance and comparison (Gámez-Guadix and Mateos-Pérez, 2026). When AI-related learning supports musical ideas, creative products, and professional self-development, burnout symptoms should be lower; when the same learning is organized around course pressure, comparison, or career anxiety, exhaustion, detachment, and reduced accomplishment should be higher.

**H1:** Autonomous interdisciplinary motivation negatively predicts learning burnout.
**H2:** Pressure-driven interdisciplinary motivation positively predicts learning burnout.

### 2.3 Perceived learning load and learning burnout

LL refers to students' appraisal of their overall learning capacity when professional music training and technology-related tasks compete for time, energy, and competence resources. Sweller (1988) linked learning difficulty to limited cognitive resources under task complexity, which provides a basis for treating load as an appraisal of demand. Later cognitive-load and subjective-effort work further connect learning load with the effort students perceive while managing instructional tasks (Sweller et al., 1998; Paas et al., 2003). For music majors, LL includes perceived pressure from practice time, rehearsal obligations, theory assignments, AI tool learning, digital creation, audio processing, feedback interpretation, and interdisciplinary course projects.

Professional music training and AI-related tasks often draw on the same limited resources. Studio practice, ensemble rehearsal, theory coursework, and project preparation already require sustained concentration and repeated revision (Bauer, 2014; Ruthmann and Mantie, 2017). AI-related learning adds software procedures, audio or MIDI editing, algorithmic-output evaluation, group coordination, and coursework that must satisfy musical and technological criteria. Generative-AI technostress and academic AI anxiety studies similarly connect digital learning with strain, confidence concerns, and continuous adaptation demands (Dizon et al., 2025; Gámez-Guadix and Mateos-Pérez, 2026). PM can intensify LL when students treat AI competence as something to acquire quickly, update continuously, and display in comparison with peers.

Maslach and Leiter (2016) located burnout in a persistent mismatch between demands and available resources, especially when learners have limited control over sustained pressure. Student burnout research describes exhaustion, detachment from learning, and reduced efficacy as related but distinguishable symptoms (Schaufeli et al., 2002; Salmela-Aro et al., 2009). LL in the present model concerns students' appraisal of time, energy, task density, and competence requirements, whereas BO concerns exhaustion, learning detachment, and reduced accomplishment. When pressure-oriented AI learning heightens perceived shortages of time, energy, and competence, learning load provides a plausible statistical link between PM and burnout.

**H3:** Perceived learning load mediates the relationship between pressure-driven interdisciplinary motivation and learning burnout.

**Figure 1:**
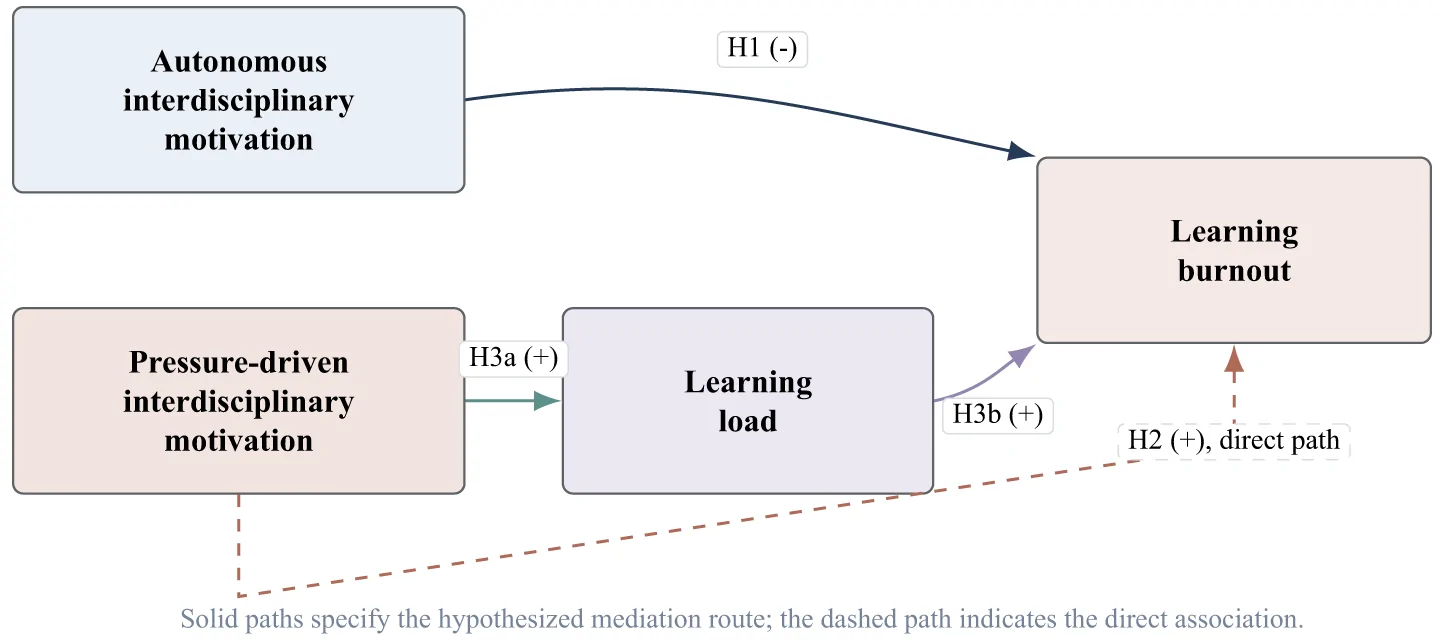
Conceptual model of interdisciplinary learning motivation, perceived learning load, and learning burnout in AI-assisted music education.

Figure 1 presents this logic as a path model. AM is specified as a direct negative predictor of BO, whereas PM is expected to relate to BO both directly and through LL, the appraisal of added time, energy, task density, and competence demands.

## 3 Methods

### 3.1 Research design and procedure

A cross-sectional questionnaire design was used to collect autonomous interdisciplinary motivation (AM), pressure-driven interdisciplinary motivation (PM), perceived learning load (LL), and learning burnout (BO) during the same survey wave. The questionnaire items were developed from the conceptual model and rewritten for learning situations familiar to university music majors. These situations included AI-assisted composition, digital arrangement, audio processing, intelligent accompaniment, automated feedback interpretation, and technology-supported teaching preparation, so the four constructs were measured through tasks that students could connect with music-major coursework and practice.

Two music education teachers reviewed the initial questionnaire for content coverage, relevance to music-major coursework, clarity of AI- and music-related terms, and comprehensibility for students with different levels of digital music experience. Before the formal survey, 15–30 music-major students completed a pilot version of the questionnaire; their responses were used for instrument refinement and were not included in the final dataset. The pilot responses helped refine completion-time estimates, item wording, scale readability, and ambiguous expressions, while also offering an initial check of internal consistency. The revised questionnaire was then administered through Wenjuanxing from March 12 to March 20, 2026.

**Figure 2:**
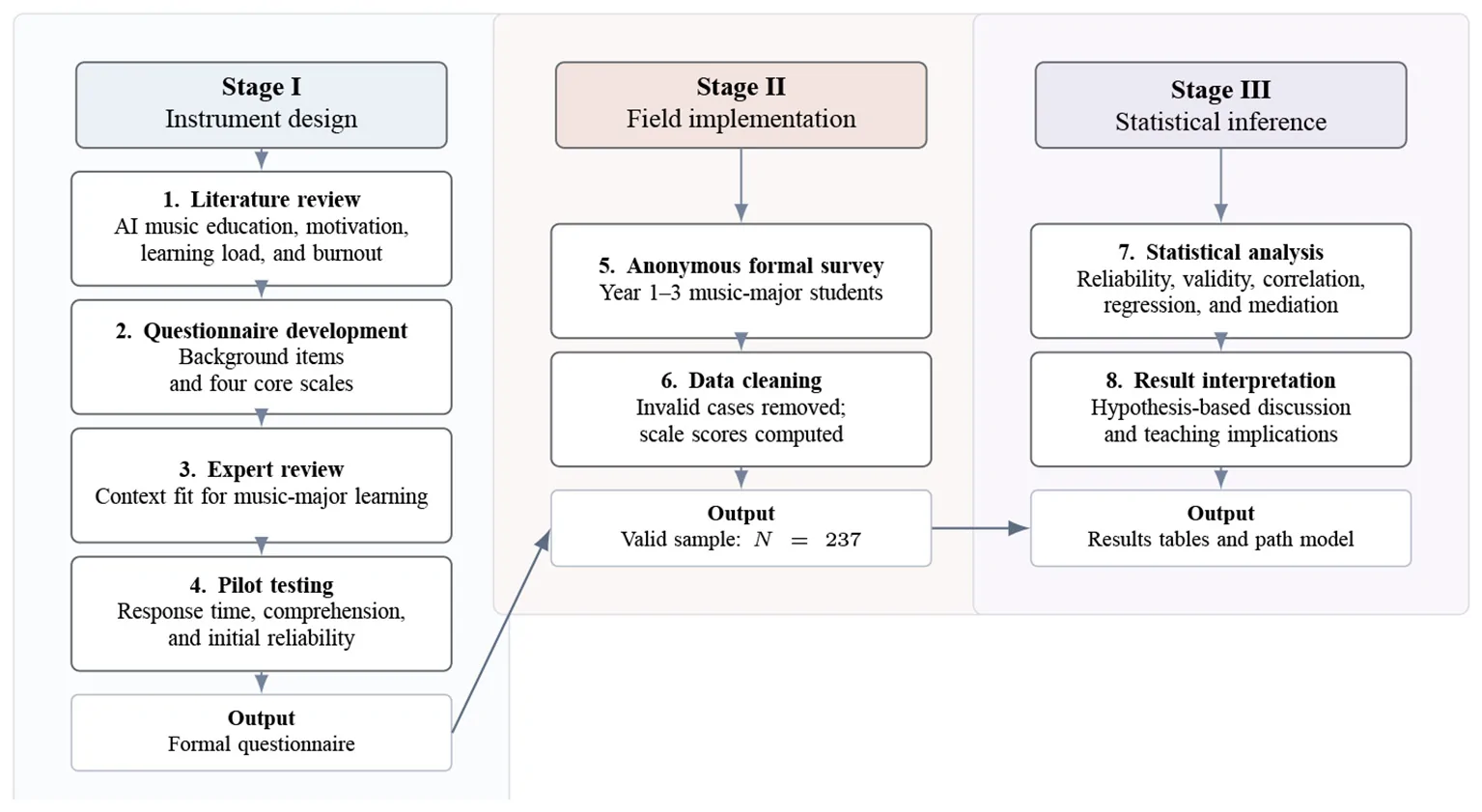
Research procedure for questionnaire design, field implementation, and statistical analysis.

Figure 2 summarizes the connection between questionnaire development, field administration, data cleaning, and regression-based hypothesis testing. It links item construction and survey implementation with the statistical tests of the AM–PM–LL–BO model.

### 3.2 Data source and sample

The formal survey was distributed through Wenjuanxing from March 12 to March 20, 2026. The study used a single-university convenience sample drawn from one music institution. Links were circulated in music theory, digital music, music education, composition-related, and performance-related courses for 1st- to 3rd-year university music majors. Course teachers helped forward the questionnaire link; they did not access individual responses or identifiable response data. Because the link moved through multiple course groups, the number of students invited directly could not be counted, and a conventional invitation-based response rate could not be calculated. Before answering, participants read a short statement explaining the research purpose, anonymous data collection, voluntary participation, and the right to withdraw.

The analytic sample included first- to third-year students enrolled in music-related majors who completed the questionnaire and provided complete data on the four core variables. AI-related course experience and AI tool-exposure frequency served as background descriptors rather than eligibility conditions. Questionnaires were excluded for non-target major or year status, incomplete submission, repeated selection of the same option, logical inconsistency, or missing data on any core variable. This screening procedure yielded 237 valid questionnaires.

**Figure 3:**
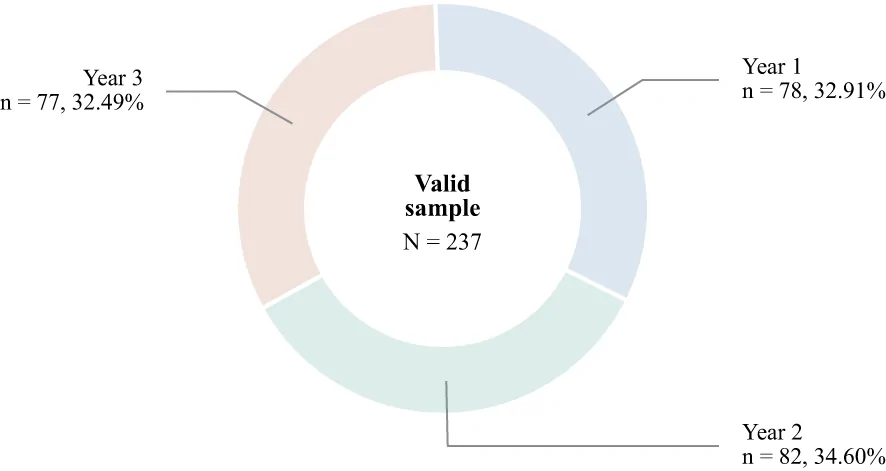
Academic year composition of the valid sample.

**Figure 4:**
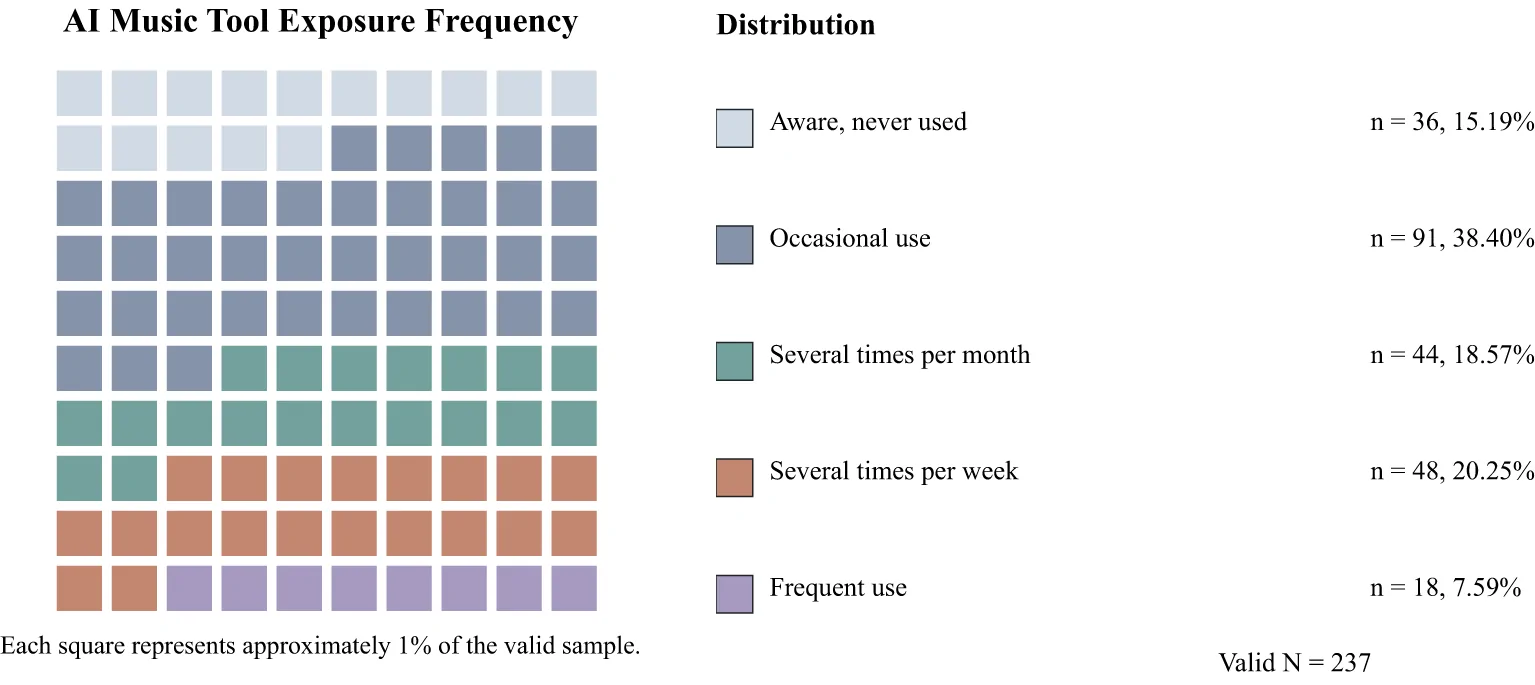
Frequency of exposure to AI-assisted music tools.

The sample structure is reported through Figures 3, 4, Table 1. Figure 3 shows 78 1st-year students (32.91%), 82 2nd-year students (34.60%), and 77 3rd-year students (32.49%), which covers early adjustment, middle-stage coursework, and advancing specialization without concentrating the sample in a single training stage. Fourth-year students were excluded because graduation performances, internships, graduation theses, graduate entrance examination preparation, and employment arrangements could introduce additional sources of learning load and burnout. Table 1 adds specialization, AI- or digital music-related course experience, and weekly professional practice or study time; the sample included performance-oriented, education-oriented, and technology-oriented music training. Among the 237 students, 102 (43.0%) had taken AI- or digital music-related courses, while 135 (57.0%) had not. Figure 4 further distinguishes awareness without use (36, 15.19%), occasional use (91, 38.40%), monthly use (44, 18.57%), weekly use (48, 20.25%), and frequent use (18, 7.59%), so the sample covers both formal course experience and informal AI exposure.

**Table 1:** Basic characteristics of the valid sample.

Variable	Category	n	Percentage
Academic year	First year	78	32.9%
	Second year	82	34.6%
	Third year	77	32.5%
Major or specialization	Music education	58	24.5%
	Vocal performance	42	17.7%
	Instrumental performance	46	19.4%
	Musicology	27	11.4%
	Composition and composition theory	18	7.6%
	Digital music	21	8.9%
	Recording arts	14	5.9%
	Music technology	11	4.6%
Taken AI/digital music-related courses	Yes	102	43.0%
	No	135	57.0%
Weekly professional practice or study time	0–5 h	24	10.1%
	6–10 h	67	28.3%
	11–15 h	75	31.6%
	16–20 h	49	20.7%
	More than 20 h	22	9.3%

### 3.3 Measures

The questionnaire used a five-point Likert scale ranging from 1 = strongly disagree to 5 = strongly agree, with higher scores indicating stronger endorsement of the measured construct. The four context-adapted measures were grounded in self-determination theory, the distinction between autonomous and pressure or control-based regulation, cognitive load and subjective-effort research, and burnout theory. AM captured internalized engagement with AI-related learning for musical expression, creative exploration, professional growth, and self-development. PM captured participation shaped by course requirements, peer comparison, employment pressure, concerns about technological replacement, and changing professional standards. LL captured students' appraisal of time, energy, task density, and competence demands when professional music training and AI- or digital music-related learning occurred together. BO captured exhaustion, detachment from learning, and reduced accomplishment during music-related and interdisciplinary study. These are context-adapted measures for the present sample, and the reliability estimates, four-factor CFA, and HTMT results reported below provide preliminary evidence for treating the four constructs as distinct in this sample. Table 2 separates motivational quality, perceived demand, and burnout symptoms so that a general attitude toward AI learning is not treated as a single construct. The full item pool in the original administration language, together with an English translation, is provided in Supplementary Table S1.

**Table 2:** Context-adapted measures, operational definitions, number of items, and example items for the core variables.

Variable	Abbrev.	Items	Operational definition and example item
Autonomous interdisciplinary motivation	AM	6	Internalized engagement in AI music, digital music, or intelligent music education because students see these areas as useful for musical expression, creative exploration, professional expansion, and self-development. Example: Even without a course requirement, I would like to use AI-assisted composition or digital arrangement to develop my own musical ideas.
Pressure-driven interdisciplinary motivation	PM	6	Engagement in AI music, digital music, or intelligent music education because of course requirements, employment uncertainty, concerns about technological replacement, peer comparison, or competitive pressure. Example: I study AI-assisted music or audio-processing tools mainly because I worry about falling behind classmates or future job requirements.
Perceived learning load	LL	6	Students' appraisal of increased demands on time, energy, task density, and competence when professional music training and AI- or digital music-related learning occur together. Example: When I have practice, rehearsal, and coursework to complete, AI or digital music tasks make my learning schedule feel overloaded.
Learning burnout	BO	8	Exhaustion, detachment from learning, and a reduced sense of accomplishment during music-related and interdisciplinary study. Example: When professional practice, coursework, and AI-related tasks overlap, I feel too exhausted to stay engaged in learning.

Mean scores were calculated because each construct was measured with multiple items on the same response scale, and averaging retained the original 1–5 metric. The mean-score calculations for student *i* are presented in Equations 1, 2:


$$\begin{array}{l} AM_{i}=\frac{1}{6}\overset{6}{\underset{j=1}{\sum }}AM_{ij},\;PM_{i}=\frac{1}{6}\overset{6}{\underset{j=1}{\sum }}PM_{ij}, \end{array}$$ (1)

$$\begin{array}{l} LL_{i}=\frac{1}{6}\overset{6}{\underset{j=1}{\sum }}LL_{ij},\;BO_{i}=\frac{1}{8}\overset{8}{\underset{j=1}{\sum }}BO_{ij}. \end{array}$$ (2)

Cronbach's α was calculated using Equation 3 to assess the internal consistency of each measure:


$$\begin{array}{l} \alpha =\frac{k}{k-1}\left(1-\frac{\sum_{j=1}^{k}\sigma_{j}^{2}}{\sigma_{X}^{2}}\right), \end{array}$$ (3)

where *k* denotes the number of items, $\sigma_{j}^{2}$ denotes the variance of item *j*, and $\sigma_{X}^{2}$ denotes the variance of the total score. Under classical measurement theory, an α value above 0.70 is generally considered acceptable (Cronbach, 1951; Nunnally and Bernstein, 1994). Internal consistency was evaluated with Cronbach's α after the four constructs had been specified theoretically and reviewed for content fit with music-major learning.

### 3.4 Statistical analysis

The statistical analysis moved from scale description to association testing. All analyses were implemented in Python 3.13.13 using numpy 2.4.6, pandas 3.0.3, scipy 1.17.1, statsmodels 0.14.6, semopy 2.3.11, factor_analyzer 0.5.1, and matplotlib 3.10.9. Descriptive statistics summarized the central tendency and dispersion of AM, PM, LL, and BO, while Cronbach's α evaluated internal consistency. Pearson correlations examined how AM and PM related to one another and how PM, LL, and BO co-varied before regression modeling. Because AM and PM are conceptually adjacent, a four-factor confirmatory factor analysis was estimated and compared with a one-factor alternative; standardized latent correlations and HTMT values served as supplementary indicators of the four constructs' empirical separability in this sample. Pearson's correlation coefficient was calculated using Equation 4:


$$\begin{array}{l} r_{XY}=\frac{\sum_{i=1}^{n}\left(X_{i}-\bar{X}\right)\left(Y_{i}-Ȳ\right)}{\sqrt{\sum_{i=1}^{n}{\left(X_{i}-\bar{X}\right)}^{2}}\sqrt{\sum_{i=1}^{n}{\left(Y_{i}-Ȳ\right)}^{2}}}. \end{array}$$ (4)

Hierarchical regression models controlled for academic year, frequency of exposure to AI-assisted tools, relevant course experience, and weekly professional learning or practice time. These covariates represent the training stage, technological exposure, course background, and the amount of time students invest in professional practice or study. The academic year was ordinally coded from first to third year, exposure frequency was ordinally coded from awareness without use to frequent use, weekly learning time was ordinally coded across five time categories, and relevant course experience was entered as a binary variable. Model 1, specified in Equation 5, examined the associations of AM and PM with BO after these covariates were included:


$$\begin{array}{l} BO_{i}=\beta_{0}+\beta_{1}Grade_{i}+\beta_{2}Freq_{i}+\beta_{3}Course_{i}+\beta_{4}Time_{i}+\beta_{5}AM_{i}\\ \;+\beta_{6}PM_{i}+\varepsilon_{i}. \end{array}$$ (5)

Model 2, specified in Equation 6, examined whether PM was associated with LL while controlling for AM and the same background variables:


$$\begin{array}{l} LL_{i}=\gamma_{0}+\gamma_{1}Grade_{i}+\gamma_{2}Freq_{i}+\gamma_{3}Course_{i}+\gamma_{4}Time_{i}+\gamma_{5}AM_{i}\\ \;+\gamma_{6}PM_{i}+\mu_{i}, \end{array}$$ (6)

$$\begin{array}{l} BO_{i}=\delta_{0}+\delta_{1}Grade_{i}+\delta_{2}Freq_{i}+\delta_{3}Course_{i}+\delta_{4}Time_{i}+\delta_{5}AM_{i}\\ \;+\delta_{6}PM_{i}+\delta_{7}LL_{i}+\eta_{i}. \end{array}$$ (7)

Model 3, specified in Equation 7, added LL to the BO regression to examine whether the PM coefficient changed after students' perceived learning load was included:


$$\begin{array}{l} IE=\gamma_{6}\times \delta_{7}. \end{array}$$ (8)

The indirect association defined in Equation 8 was tested using 5,000 Bootstrap resamples, with significance evaluated by whether the 95% confidence interval excluded zero (Preacher and Hayes, 2008; Hayes, 2018). Before interpreting the regression models, the analysis inspected the suitability of the model estimates through checks for multicollinearity, residual distribution, and influential observations. Anonymous responses, mixed item ordering, and the absence of right or wrong answer cues were used to reduce common method bias (Podsakoff et al., 2003; Kline, 2016). Because the survey was cross-sectional, the mediation model estimated a regression-based indirect association among PM, LL, and BO.

## 4 Results

### 4.1 Descriptive statistics, internal consistency, and correlations

Table 3 summarizes reliability, descriptive statistics, and correlations for the four core variables before the regression models. Cronbach's α values ranged from 0.822 to 0.882, indicating acceptable internal consistency for the four context-adapted measures. AM had the highest mean (*M* = 3.592), showing relatively strong endorsement of self-directed AI-related learning in the sample. BO had the lowest mean (*M* = 2.972), while its standard deviation showed enough individual variation for regression analysis.

**Table 3:** Descriptive statistics, internal consistency, and correlation matrix for the core variables.

Variable	M	SD	α	1	2	3	4
1. Autonomous interdisciplinary motivation	3.592	0.586	0.822	1	0.010	−0.213^***^	−0.265^***^
2. Pressure-driven interdisciplinary motivation	3.266	0.606	0.825	0.010	1	0.442^***^	0.415^***^
3. Perceived learning load	3.172	0.628	0.849	−0.213^***^	0.442^***^	1	0.551^***^
4. Learning burnout	2.972	0.637	0.882	−0.265^***^	0.415^***^	0.551^***^	1

^***^p < 0.001.

AM and PM were nearly uncorrelated (*r* = 0.010), indicating that students' autonomous and pressure-driven reasons for AI-related learning varied independently in this sample. The AM items emphasize interest, value, and self-development, whereas the PM items emphasize concern, comparison, and falling behind, so the near-zero correlation should be read as empirical separation in this sample, with item-valence differences kept in mind. AM was negatively correlated with LL (*r* = −0.213, *p* < 0.001) and BO (*r* = −0.265, *p* < 0.001), whereas PM was positively correlated with LL (*r* = 0.442, *p* < 0.001) and BO (*r* = 0.415, *p* < 0.001). The LL–BO correlation was the largest in the matrix (*r* = 0.551, *p* < 0.001), supporting the planned regression test of the PM–LL–BO path.

Because AM and PM are conceptually adjacent, a four-factor confirmatory factor analysis was estimated before the regression models were interpreted. The four-factor solution fit well (CFI = 0.986, TLI = 0.985, RMSEA = 0.021, χ^2^ *p* = 0.103), whereas a one-factor alternative fit poorly (CFI = 0.618, RMSEA = 0.110). The contrast between the two models indicates that a single undifferentiated factor cannot explain the covariance pattern in this sample, while the shared-source design still leaves room for common-method influence. The standardized AM-PM latent correlation was 0.009, and HTMT values ranged from 0.134 to 0.637, which points to a preliminary empirical distinction between the two motivation scales in this sample.

**Figure 5:**
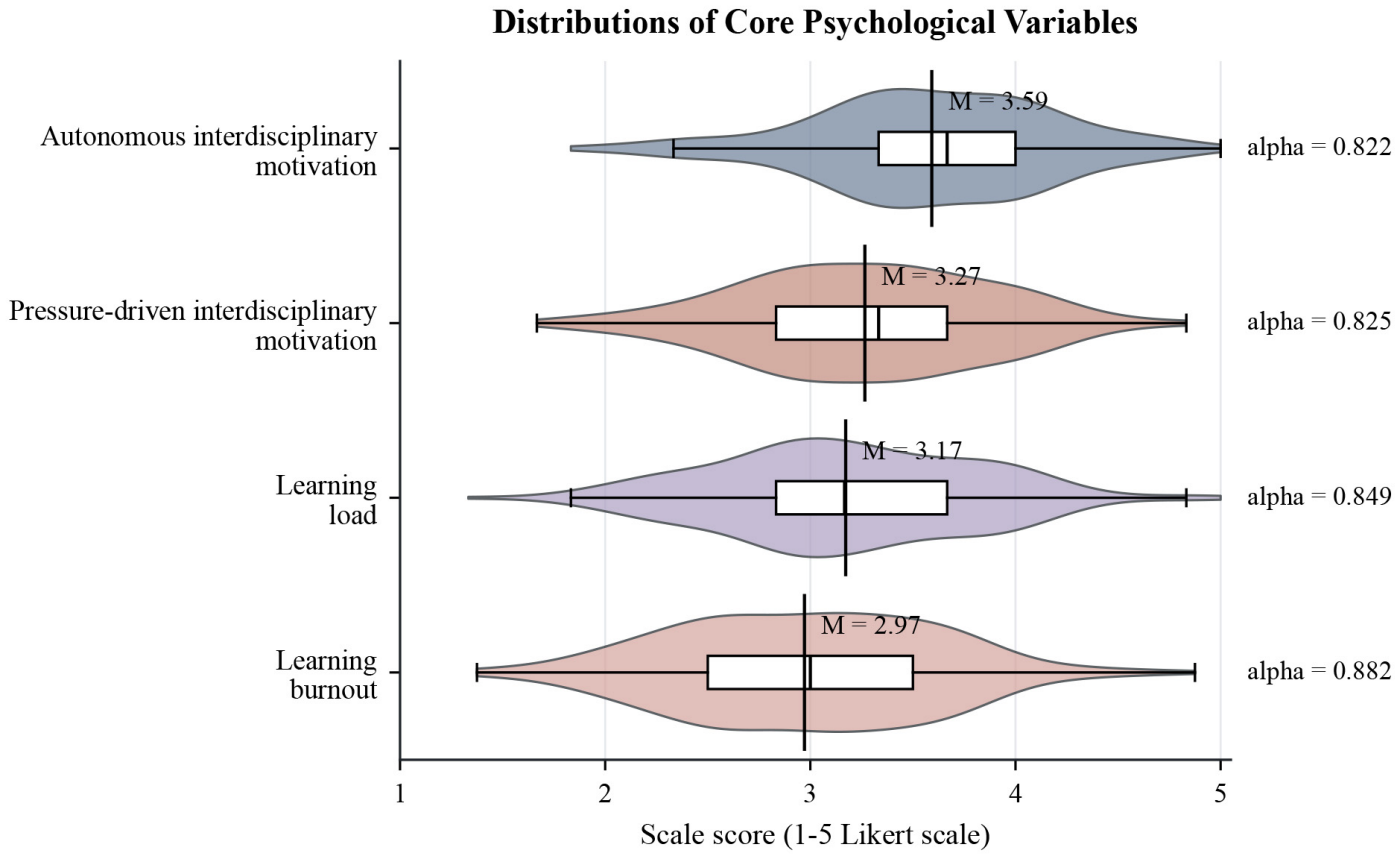
Distributions of the four core psychological variables. The horizontal axis represents mean scale scores on a 1–5 Likert scale, ranging from 1 = strongly disagree to 5 = strongly agree, while the vertical axis lists the four psychological variables. The violin shapes show the score distributions, the embedded boxplots show the medians and interquartile ranges, the vertical lines indicate the means, and Cronbach's α coefficients are displayed on the right.

Figure 5 shows whether the scale scores had enough spread for the planned regression analysis. AM was concentrated toward the upper part of the scale, while PM, LL, and BO were closer to the scale midpoint. The visible interquartile ranges and continuous distributions reduce concern that the regression estimates were driven by highly restricted responses, especially for PM and LL, the variables central to the planned PM–LL–BO path.

### 4.2 Regression and hypothesis testing

Table 4 organizes the hypothesis tests across three regression models. M1 examined whether AM and PM were associated with BO after controlling for academic year, exposure frequency, relevant course experience, and weekly professional learning time. AM was associated with lower BO (*B* = −0.285, β = −0.262, *p* < 0.001), whereas PM was associated with higher BO (*B* = 0.430, β = 0.408, *p* < 0.001). The opposite signs match the directions stated in H1 and H2.

**Table 4:** Hierarchical regression and Bootstrap indirect-effect analysis.

Model and path	B	SE	β	p	*R* ^2^	95% CI
M1: Academic year → Learning burnout	0.068	0.045	0.087	0.134	0.254	[–0.021, 0.158]
M1: Exposure frequency → Learning burnout	0.018	0.032	0.033	0.572	0.254	[–0.044, 0.080]
M1: Relevant course experience → Learning burnout	0.044	0.076	0.034	0.562	0.254	[–0.105, 0.193]
M1: Weekly professional learning time → Learning burnout	0.002	0.032	0.003	0.955	0.254	[–0.062, 0.066]
M1: Autonomous interdisciplinary motivation → Learning burnout	-0.285	0.063	-0.262	< 0.001	0.254	[-0.409, -0.161]
M1: Pressure-driven interdisciplinary motivation → Learning burnout	0.430	0.060	0.408	< 0.001	0.254	[0.311, 0.548]
M2: Academic year → Perceived learning load	0.097	0.044	0.125	0.031	0.262	[0.009, 0.184]
M2: Exposure frequency → Perceived learning load	-0.033	0.031	-0.061	0.293	0.262	[–0.094, 0.028]
M2: Relevant course experience → Perceived learning load	0.026	0.074	0.020	0.730	0.262	[–0.121, 0.172]
M2: Weekly professional learning time → Perceived learning load	0.010	0.032	0.018	0.746	0.262	[–0.052, 0.073]
M2: Autonomous interdisciplinary motivation → Perceived learning load	–0.221	0.062	–0.206	< 0.001	0.262	[–0.342, –0.100]
M2: Pressure-driven interdisciplinary motivation → Perceived learning load	0.454	0.059	0.438	< −.001	0.262	[0.338, 0.570]
M3: Academic year → Learning burnout	0.028	0.042	0.036	0.502	0.376	[–0.054, 0.111]
M3: Exposure frequency → Learning burnout	0.031	0.029	0.058	0.281	0.376	[–0.026, 0.089]
M3: Relevant course experience → Learning burnout	0.033	0.069	0.026	0.631	0.376	[–0.103, 0.170]
M3: Weekly professional learning time → Learning burnout	-0.002	0.030	-0.004	0.935	0.376	[–0.061, 0.056]
M3: Autonomous interdisciplinary motivation → Learning burnout	–0.194	0.059	–0.178	0.001	0.376	[–0.310, –0.077]
M3: Pressure-driven interdisciplinary motivation → Learning burnout	0.242	0.062	0.230	< 0.001	0.376	[0.120, 0.364]
M3: Perceived learning load → Learning burnout	0.413	0.062	0.408	< 0.001	0.376	[0.292, 0.535]
Bootstrap indirect effect: PM → LL → BO	0.188	0.037	–	–	–	[0.122, 0.264]

All regression models controlled for academic year, frequency of exposure to AI-assisted tools, relevant course experience, and weekly professional learning time. The Bootstrap row reports a cross-sectional indirect effect.

M2 tested whether PM was associated with LL, the proposed mediator. PM was associated with higher LL (*B* = 0.454, β = 0.438, *p* < 0.001), indicating that pressure-driven engagement corresponded to stronger perceived demands on time, energy, task density, and competence. AM was associated with lower LL (*B* = −0.221, β = −0.206, *p* < 0.001). Among the control variables, only academic year was associated with LL in M2 (*B* = 0.097, *p* = 0.031); the remaining controls were non-significant across the three models.

M3 added LL to the BO regression and increased the explained variance from 0.254 to 0.376. LL was associated with BO (*B* = 0.413, β = 0.408, *p* < 0.001). The coefficient for PM remained significant, but its standardized value decreased from 0.408 to 0.230 (*B* = 0.242, *p* < 0.001). The Bootstrap indirect effect was *B* = 0.188, with a 95% confidence interval of [0.122, 0.264]. The nonzero Bootstrap interval and the remaining PM coefficient indicate partial mediation in the regression sense: in this cross-sectional sample, LL statistically accounted for part of the association between PM and BO.

**Figure 6:**
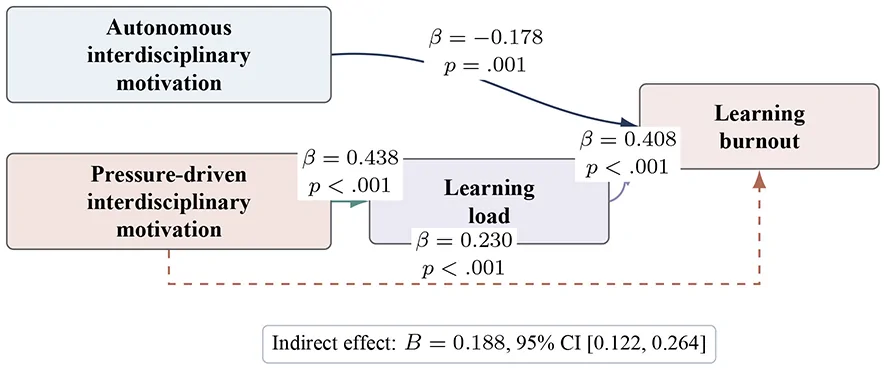
Standardized mediation path model of pressure-driven motivation, perceived learning load, and learning burnout.

Figure 6 displays the standardized paths corresponding to the cross-sectional indirect-effect test. The PM → LL and LL → BO paths were both significant, and the PM → BO path remained significant after LL was added.

## 5 Discussion

AM and PM emerged as distinct sources of participation, with AM aligned to lower BO and PM aligned to higher LL and BO. Their observed and latent correlations were close to zero, so the two motivational sources carried different psychological meanings for AI-related learning. AI work in this setting connected technology with musical aims for some students and with the need to keep pace with external demands for others.

Self-determination theory helps explain why AM is aligned with lower BO (Ryan and Deci, 2000, 2017). When students use AI to test timbre, draft an arrangement, shape accompaniment, compare compositional ideas, revise audio, or prepare teaching materials, the technology serves a musical purpose that they recognize as their own. Those tasks give students something concrete to refine, hear, and present, and that cycle strengthens task value, progress, and ownership. AI learning then supports professional autonomy and a clearer sense of accomplishment.

PM aligned with higher LL and BO because pressure-oriented engagement keeps students active while changing the meaning of the activity. Technology acceptance research shows that perceived usefulness and social influence support adoption (Davis, 1989; Venkatesh et al., 2003), and in music programs, those forces meet course evaluation, peer comparison, internship preparation, employment competition, technological replacement concerns, and changing professional standards. Students already manage practice, rehearsal, performance, theory coursework, and pedagogy, so AI competence enters an already demanding training structure. Recent work on generative-AI anxiety, ethical anxiety, and fear of AI shows how competence concern and future standing can shape this kind of participation (Zhan et al., 2023; Zhu et al., 2025; Cengiz and Peker, 2025; Gámez-Guadix and Mateos-Pérez, 2026); effort remains high, but strain rises with it.

LL explains part of the PM–BO association because pressure-oriented participation changes how students read the demands placed on them. AI learning adds software procedures, audio or MIDI editing, algorithmic-output evaluation, group coordination, and interdisciplinary coursework to practice time, rehearsal obligations, theory assignments, and teaching preparation. Time and energy then feel scarce, task density rises, and competence requirements appear more diffuse, and those appraisals correspond to exhaustion, learning detachment, and weaker accomplishment. The remaining direct PM–BO association keeps attention on evaluation anxiety, uncertainty about digital competence, comparison with classmates, and tension between traditional musician identity and new digital standards.

These findings point to a practical course-design principle: AI tasks work best when they are organized around musical outcomes. Tool operation can be taught alongside musical judgment, creative decision-making, and reflective explanation, with each stage carrying a different level of difficulty. A student who is learning sound editing, accompaniment generation, or AI-assisted composition should know whether the task is meant to practice software control, refine a musical idea, or explain a pedagogical choice. Assessment that separates those elements reduces pressure to master every platform at once and keeps AI learning tied to composition, arrangement, listening analysis, teaching preparation, and reflective review.

The four measures were developed for AI-assisted music learning, and the internal consistency, four-factor CFA, and HTMT results support their distinctiveness in this dataset. LL and BO also share wording around overload, emotional exhaustion, and difficulty sustaining engagement, which helps explain their correlation. The questionnaire data came from one university and one wave of measurement, so replication across institutions and longitudinal observation would clarify the scale structure and the development of the PM, LL, and BO relationships over time.

## 6 Conclusion

Among 237 1st- to 3rd-year university music majors from a single university, autonomous, and pressure-driven sources of AI-related interdisciplinary learning motivation showed different associations with learning burnout. AM was associated with lower BO, whereas PM was associated with higher LL and BO, and the Bootstrap test showed a cross-sectional indirect association from PM to BO through LL. These findings rest on single-source, cross-sectional data from one institution, and the context-adapted measures still need replication in independent samples. For music majors, AI-related learning should be organized around musical purposes–composition, arrangement, listening analysis, teaching preparation, and reflective evaluation–with technical tasks serving professional growth and competitive technical requirements kept subordinate to those musical purposes.

## Data Availability

The raw data supporting the conclusions of this article will be made available by the authors, without undue reservation.
