# Supplementary material for: The relationship between interdisciplinary learning motivation and learning burnout among university music majors in the context of AI-assisted music education: the mediating role of perceived learning load
Source: Front Psychol. 2026 Aug 7;17:1922731. doi: 10.3389/fpsyg.2026.1922731 (PMC13493522; doi:10.3389/fpsyg.2026.1922731)
Supplement: Supplementary file 1 [file Data_Sheet_1.pdf]

Supplementary Material

1 SUPPLEMENTARY TABLE S1. FULL ITEM POOL

All items were scored on a 5-point Likert scale ranging from 1 = strongly disagree to 5 = strongly agree. The English wording below is an author translation of the original Chinese administration version.

Autonomous interdisciplinary motivation (AM)

Code	Original Chinese item	English translation
AM1	我对 AI 音乐、数字音乐或智能音乐教学相关内容感兴趣。	I am interested in AI music, digital music, or intelligent music education content.
AM2	我愿意主动了解 AI 技术在音乐创作或音乐教育中的应用。	I am willing to actively learn about applications of AI technology in music creation or music education.
AM3	我认为跨学科学习可以拓展我的音乐表达方式。	I believe interdisciplinary learning can expand my ways of musical expression.
AM4	我学习 AI 或数字音乐相关内容，是因为它能帮助我形成新的专业能力。	I learn AI- or digital music-related content because it helps me develop new professional skills.
AM5	即使没有课程要求，我也愿意接触 AI 与音乐结合的相关内容。	Even without course requirements, I am willing to engage with content that combines AI and music.
AM6	我愿意尝试将 AI 工具用于音乐创作、音乐教学设计或作品分析。	I am willing to try using AI tools for music composition, music teaching design, or work analysis.

Pressure-driven interdisciplinary motivation (PM)

Code	Original Chinese item	English translation
PM1	我学习 AI 或数字音乐相关内容，主要是因为担心以后缺乏竞争力。	I study AI- or digital music-related content mainly because I worry that I may lack competitiveness in the future.

Code	Original Chinese item	English translation
PM2	看到同学学习 AI 音乐工具，我会觉得自己也必须跟上。	When I see classmates learning AI music tools, I feel that I must keep up.
PM3	我担心如果不掌握 AI 或数字音乐技术，未来发展会受到影响。	I worry that if I do not master AI or digital music technology, my future development will be affected.
PM4	我接触 AI 音乐相关内容，更多是出于压力，而不是兴趣。	I engage with AI music-related content more out of pressure than interest.
PM5	我觉得 AI 技术的发展让我对音乐专业未来产生一定不安。	I feel somewhat uneasy about the future of the music profession because of the development of AI technology.
PM6	我有时担心传统音乐训练不足以应对未来音乐教育或音乐行业的变化。	I sometimes worry that traditional music training will not be enough to cope with changes in future music education or the music industry.

Perceived learning load (LL)

Code	Original Chinese item	English translation
LL1	在原有专业训练之外学习 AI 或数字音乐内容，会让我感到负担增加。	Learning AI or digital music content in addition to my existing professional training makes me feel more burdened.
LL2	我觉得音乐专业课程已经较多，再加入技术类内容会带来压力。	I feel that music-major coursework is already heavy, and adding technology-related content creates pressure.
LL3	AI 或数字音乐相关学习常常占用我的专业练习时间。	AI- or digital music-related learning often takes up my practice time.
LL4	当课程同时要求音乐能力和技术能力时，我容易感到吃力。	I find it difficult when courses require both musical ability and technical ability at the same time.
LL5	我有时觉得自己难以同时兼顾传统专业训练和跨学科学习。	I sometimes find it difficult to balance traditional professional training and interdisciplinary learning at the same time.

Code	Original Chinese item	English translation
LL6	面对新的音乐技术学习任务时，我常感到时间和精力不够。	When faced with new music-technology learning tasks, I often feel short of time and energy.

Learning burnout (BO)

Code	Original Chinese item	English translation
BO1	最近一段时间，我对专业学习容易感到疲惫。	Recently, I have often felt tired of my professional study.
BO2	面对专业课程和跨学科学习任务时，我有时感到精力不足。	When facing professional courses and interdisciplinary learning tasks, I sometimes feel low on energy.
BO3	我会觉得持续投入专业学习越来越吃力。	I feel that it is becoming increasingly difficult to keep investing in my professional study.
BO4	学习 AI 或数字音乐相关内容时，我有时会觉得自己难以继续保持热情。	When studying AI- or digital music-related content, I sometimes feel unable to keep up my enthusiasm.
BO5	即使完成了学习任务，我也常觉得收获感不强。	Even when I complete learning tasks, I often feel that my sense of achievement is weak.
BO6	面对频繁的专业训练和课程任务，我容易产生想要退开一点的感觉。	Faced with frequent professional practice and coursework, I easily feel like pulling back a little.
BO7	我觉得自己在音乐专业学习中越来越难获得满足感。	I feel that it is becoming harder and harder to gain satisfaction from my music-major study.
BO8	专业训练和跨学科学习叠加时，我常觉得自己被学习耗得很空。	When professional training and interdisciplinary learning overlap, I often feel drained by learning.

2 MEASUREMENT NOTE

The main text reports the four-factor confirmatory factor analysis and discriminant-validity indices used to examine the distinctiveness of AM, PM, LL, and BO.
